# Chronic pain as a risk factor for Alzheimer’s disease pathology: a longitudinal ADNI study

**DOI:** 10.1186/s13195-026-02137-x

**Published:** 2026-07-03

**Authors:** Tyler R. Bell, Carol E. Franz, Christine Fennema-Notestine, Erin E. Sundermann, Shelby B. Hughes, Matthew S. Panizzon, Imanuel Lerman, Jeremy A. Elman, William S. Kremen

**Affiliations:** 1 Department of Psychiatry, University of California San Diego, 9500 Gilman Drive MC-0738, La Jolla, CA 92093 USA; 2 Center for Behavior Genetics of Aging, University of California, San Diego, La Jolla, CA 92093 USA; 3 Department of Radiology, University of California San Diego, La Jolla, CA 92093 USA; 4 Department of Anesthesiology, University of California, San Diego, La Jolla, CA 92093 USA

**Keywords:** Chronic pain, Alzheimer’s disease, Cognition, APOE-ε4, Amyloid-β, p-tau181, Longitudinal MRI, Alzheimer’s disease neuroimaging initiative (ADNI)

## Abstract

**Background:**

Growing evidence supports chronic pain (CP) as a risk factor for Alzheimer’s disease and Alzheimer’s disease-related dementias (AD/ADRD). We examined whether CP is associated with longitudinal worsening of AD-related biomarkers, brain structure, and cognition, and whether effects differ by APOE-ε4 status.

**Methods:**

Data were drawn from 1,493 participants in the Alzheimer’s Disease Neuroimaging Initiative (ADNI) without baseline dementia (mean age = 72.4 ± 7.19 years; 48% female). Linear mixed-effects models tested longitudinal change in cerebrospinal fluid (CSF) and positron emission tomography (PET) amyloid-β (Aβ) and phosphorylated tau (p-tau181), structural MRI indices (AD-related cortical signature, hippocampal and entorhinal volumes, precuneus thickness), and cognitive outcomes (episodic memory, executive function, Mini-Mental State Examination [MMSE]). Models included fixed effects of CP status, APOE-ε4 status, time (years since baseline), and their two-way and three-way interactions, adjusting for age, sex, education, baseline Aβ positivity status, and time-varying depressive symptoms.

**Results:**

At baseline, 30% of participants (*n* = 452) reported CP and 45% (*n* = 666) were APOE-ε4 carriers. CP and APOE-ε4 status did not independently predict baseline CSF Aβ42/Aβ40, PET Aβ burden, or CSF p-tau181. Longitudinally, the CP+/ε4 + group showed greater AD-like changes in CSF Aβ42/Aβ40 and CSF p-tau181, thinning of the precuneus (β=-0.09, *p*<.001) and faster decline in episodic memory (β=-0.08, *p*<.001), compared to all other groups. Many of these associations remained when excluding participants with MCI or restricting to Aβ-negative participants.

**Conclusions:**

The combination of CP and APOE-ε4 carriership was associated with greater longitudinal worsening of brain structure and cognitive function, even among individuals who were Aβ-negative at baseline. These findings provide in vivo evidence that CP is associated with greater AD-related worsening over time in the context of genetic susceptibility. As evidence accumulates, CP may warrant recognition as a risk factor in dementia prevention frameworks, though causal inferences require further study.

**Supplementary Information:**

The online version contains supplementary material available at https://doi.org/10.1186/s13195-026-02137-x.

## Background

Chronic pain (CP) — defined as pain that persists or recurs for more than 3 to 6 months — affects nearly 1 in 5 older adults and has emerged as a risk factor for Alzheimer’s disease and Alzheimer’s disease-related dementias (AD/ADRD) [1]. Large-scale epidemiological studies suggest that CP is associated with greater cognitive decline over time and significantly increases the risk for incident cognitive impairment and AD/ADRD [2, 3]. For example, Khalid et al. reported that individuals with CP were more than twice as likely to develop dementia compared to those without CP (HR = 2.77) [2]. However, CP is not included in the 2024 Lancet report on risk factors for dementia, highlighting the need for further study and additional evidence on the association of CP with AD-related biomarkers, neurodegeneration, and cognitive decline [4].

Most causal evidence linking CP to AD pathology comes from preclinical rodent studies, which have shown that the induction of CP leads to increased accumulation of beta-amyloid (Aβ) and tau proteins—key components of amyloid plaques and neurofibrillary tangles, the hallmark pathologies of AD [5, 6]. Human studies are needed to generalize this relationship. Clinical studies that include highly profiled individuals with data on cognition, brain, and AD-related biomarkers and longitudinal follow-up, are thus an important source for examining this issue.

One of the first major human studies to explore the relationship between CP and AD-related biomarkers was conducted by Sadlon et al. [7] Using data from the Alzheimer’s Disease Neuroimaging Initiative (ADNI), they examined how CP relates to biomarker and cognitive change over time. Analyses were stratified by baseline ATN (amyloid [Aβ], tau, neurodegeneration) profiles, regardless of clinical diagnosis (cognitively unimpaired, mild cognitive impairment [MCI], or dementia). They found that, at baseline, individuals with CP in an A–/TN+ group (suspected non-Alzheimer disease pathophysiology) had increasing levels of cerebrospinal fluid (CSF) total tau over time. Among individuals who were completely biomarker negative at baseline (A–/TN–), CP predicted increases in CSF phosphorylated tau 181 (p-tau181) over time. However, they did not find associations of CP with longitudinal cognitive decline within any A/TN group. It is notable that mean Clinical Dementia Rating-Sum of Boxes was 1.86 and 1.53 in the non-pain and pain groups, respectively [7]. These numbers suggest a large proportion of dementia cases, which precludes assessing pain as a risk factor. In addition, they did not examine the role of APOE-ε4, the largest genetic risk factor for AD pathology.

Our work thus poses a distinct question: Is CP associated with greater accumulation of AD-related biomarkers as well as neurodegeneration and cognitive decline over time? The present study extends our earlier findings from the Religious Orders Study and Memory and Aging Project (ROSMAP), where CP was classified prior to dementia onset to evaluate its role as a potential risk factor [8]. In that study, older adults with CP had greater cognitive decline and greater post-mortem Aβ accumulation than those without CP, but only among APOE-ε4 carriers. Leveraging the ADNI dataset, we extend this work by examining how CP is associated with longitudinal changes in in vivo AD-related biomarkers, brain structure, and cognition. Because our prior ROSMAP findings highlighted the important role of APOE-ε4, in the present study we examined both the independent associations of CP with AD-related outcomes and its interaction with APOE-ε4 status, hypothesizing that the combination of CP and APOE-ε4 carriership would be associated with the greatest longitudinal worsening. For sensitivity analyses, we examined how associations differed when restricting the sample to those who were cognitively unimpaired or Aβ-negative at baseline.

## Methods

### Participants

Data used in the preparation of this article were obtained from the Alzheimer’s Disease Neuroimaging Initiative (ADNI) database (adni.loni.usc.edu). ADNI was launched in 2003 as a public-private partnership, led by Principal Investigator Michael W. Weiner, MD. The primary goal of ADNI has been to test whether serial magnetic resonance imaging (MRI), positron emission tomography (PET), other biological markers, and clinical and neuropsychological assessment can be combined to measure the progression of MCI and early AD. For up-to-date information, see www.adni-info.org. The study was organized into multiple phases of data collection occurring across academic and medical institutions in the United States and Canada: ADNI1 (2004–2010), ADNIGO (2009–2011), ADNI2 (2011–2016), and ADNI3 (2016–2022). Each phase included participants who were cognitively unimpaired, had MCI, and those with dementia. All phases assessed changes in cognition, fluid biomarkers, and neuroimaging measures. All study procedures adhered to the Declaration of Helsinki, with institutional review board approval obtained from all participating institutions. Written informed consent was provided by all participants.

For the present study, we limited data to ADNI individuals with medical history interviews where they could report CP (*n* = 4088). Individuals diagnosed with dementia at baseline were excluded to focus on baseline CP as a risk factor for dementia (*n* = 633); however, we retained those who developed dementia during follow-up assessments. To remove possible confounding from medication use, we excluded individuals who reported opioid use (*n* = 25). We also removed individuals who did not report CP at baseline but developed it later (*n* = 94). We additionally removed individuals with no information on APOE-ε4 status and insufficient information to determine baseline Aβ status (*n* = 1,843). This led to a final sample of 1,493 individuals, including participants from ADNI1 (*n* = 130), ADNIGO (*n* = 127), ADNI2 (*n* = 763), and ADNI3 (*n* = 473). To maximize statistical power, all available follow-ups for each individual were included. Participant characteristics, along with details on selected outcomes and covariates, are provided in Table 1. All ADNI data were provided from the Image and Data Archive (IDA, https://ida.loni.usc.edu).

**Table 1:** Baseline participant characteristics by chronic pain and APOE ε4 Status

Characteristic	Statistic	Overall (*n* = 1493)	CP-/ε4- (*n* = 573)	CP-/ε4+ (*n* = 468)	CP+/ε4- (*n* = 254)	CP+/ε4+ (*n* = 198)	F/χ²	*p*	*n*
Age	M (SD)	72.4 (7.19)	72.6 (7.24)	71.3 (6.98)	73.9 (7.15)	72.5 (7.23)	7.60	< .001^ad^	1493
Education (Years)	M (SD)	16.3 (2.54)	16.6 (2.49)	16.2 (2.49)	16.2 (2.62)	16.1 (2.69)	3.32	0.019	1493
Sex							0.25	0.969	1493
Male	n (%)	770 (52%)	300 (52%)	239 (51%)	129 (51%)	102 (52%)			
Female	n (%)	723 (48%)	273 (48%)	229 (49%)	125 (49%)	96 (48%)			
Depressive Symptoms	M (SD)	1.4 (1.42)	1.2 (1.42)	1.4 (1.39)	1.5 (1.47)	1.5 (1.44)	3.99	.008^b^	1493
MCI Status							62.41	< .001^abcef^	1270
Yes	n (%)	658 (52%)	209 (40%)	205 (58%)	129 (56%)	115 (73%)			
No	n (%)	612 (48%)	313 (60%)	153 (42%)	103 (44%)	43 (27%)			
Aβ Positivity							126.67	< .001_abcdf_	1493
No	n (%)	603 (40%)	261 (46%)	119 (25%)	166 (65%)	57 (29%)			
Yes	n (%)	890 (60%)	312 (54%)	349 (75%)	88 (35%)	141 (71%)			
Tau Positivity							87.33	< .001^ac^	622
Yes	n (%)	218 (35%)	56 (18%)	127 (53%)	11 (32%)	24 (63%)			
No	n (%)	404 (65%)	255 (82%)	112 (47%)	23 (68%)	14 (37%)			

*CP* Chronic pain, *ε4* APOE ε4 allele, *M* Mean, *SD* Standard deviation, *MCI* Mild cognitive impairment, *MMSE* Mini-Mental State Examination, *AD* Alzheimer’s disease

Superscripts denote significant post-hoc pairwise group differences (p <. 05): ^a^ CP-/ε4- vs. CP-/ε4+; ^b^ CP-/ε4- vs. CP+/ε4-; ^c^ CP-/ε4- vs. CP+/ε4+; ^d^ CP-/ε4 + vs. CP+/ε4-; ^e^ CP-/ε4 + vs. CP+/ε4+; ^f^ CP+/ε4- vs. CP+/ε4+. Tukey’s HSD and proportion pairwise tests were used for continuous variables and categorical variables, respectively

The ADNI protocol was approved by the institutional review boards of all participating sites, and the study was conducted in accordance with the Declaration of Helsinki and its later amendments. Written informed consent was obtained from all participants or their authorized representatives. We considered disease heterogeneity by stratifying analyses on APOE-ε4 status and baseline MCI, and by adjusting for demographic covariates (age, sex, education).

## Measures

### Chronic pain (CP)

Participants were asked at each visit about the presence of any health symptoms (data in the *recblog* and *recmhis* files accessed October 31, 2024). They were further asked about the date of onset for the symptom, the chronicity of the symptom (once occurring, persistent, or intermittent). From these data we examined symptoms of musculoskeletal pain – the most common pain type in older adults [8]. Consistent with the definition of pain from the International Association for the Study of Pain (IASP), we defined CP as pain reported as persistent or intermittent with an onset greater than 3 months prior [9].

### CSF biomarkers

CSF Aβ and tau biomarker data were obtained from the ADNI Biomarker Core Laboratory at the University of Pennsylvania and downloaded from the ADNI database (*UPENNBIOMK12_2020* file, accessed March 1, 2026). CSF samples were collected by lumbar puncture following standardized ADNI pre-analytical protocols, processed into 0.5 mL aliquots, and stored at − 80 °C until analysis. On the day of analysis, thawed CSF samples were analyzed in singlicate for Aβ42, Aβ40, and p-tau181 using Roche Elecsys electrochemiluminescence immunoassays (Elecsys β-Amyloid(1–42) CSF, Elecsys β-Amyloid (1–40) CSF, and Elecsys Phospho-Tau(181P) CSF) on the fully automated cobas e 601 analyzer (Roche Diagnostics) [10]. The CSF Aβ42/Aβ40 ratio and CSF p-tau181 were used as primary CSF outcomes. Cutoffs to determine Aβ and tau positivity are discussed below and were validated against PET data.

### PET biomarkers

PET Aβ imaging data were obtained from the ADNI database (*ADSP_PHC_PET_Amyloid_Detailed* file, accessed March 1, 2026) and were generated by the Alzheimer’s Disease Sequencing Project - Phenotype Harmonization Consortium (ADSP-PHC). We focused on PET scans using either Florbetaben (FBB) or Florbetapir (FBP), which were acquired in 5-minute frames between 90 and 110 min post-injection for FBB, and between 50 and 70 min post-injection for FBP. These tracers have been validated for their ability to distinguish individuals with AD dementia from cognitively unimpaired individuals and correlate strongly with postmortem Aβ pathology [11, 12].

The primary measure of Aβ burden was the pre-calculated centiloid variable provided by the ADSP-PHC, derived using the GAAIN atlas-based SUVR pipeline and the ADSP-PHC-recommended centiloid conversion equations. All quantification, conversion equations, and positivity thresholds were taken from the same ADSP-PHC GAAIN atlas pipeline to ensure internal consistency. Aβ positivity thresholds and centiloid conversion equations followed those documented in the ADSP-PHC methods documentation (ADNI_ADSP_PHC_PET_AMYLOID_TAU_METHODS_20250217.pdf).

PET tau data was also derived by the ADSP-PHC and downloaded from the ADNI database (*ADSP_PHC_PET_Tau_Detailed* file, accessed March 1, 2026). Tau imaging used the tracer [¹⁸F] Flortaucipir (FTP, also known as AV-1451 or T807), which binds selectively to tau protein aggregates. FTP PET scans were acquired between 75 and 105 min post-injection. Elevated cortical binding of FTP has been associated with clinical impairment and cognitive decline, particularly in regions such as the inferior temporal gyrus, and the tracer is widely used to stage tau pathology and monitor disease progression in AD [13].

Tau SUVRs were normalized to the inferior cerebellum and summarized for regions associated with Braak stages B1/B2, B3, B4, B5, and B6. Detailed methods for PET preprocessing, image coregistration, and harmonization procedures are described in the ADNI documentation (*ADNI_ADSP_PHC_PET_AMYLOID_TAU_METHODS_20250217.pdf*). In our sample, the number of PET tau scans was limited across CP and APOE-ε4 subgroups; therefore, PET tau was not included as a primary study outcome. Instead, PET tau data were used solely to define baseline tau positivity, which was included as a covariate in sensitivity analyses as described below.

### MRI

Structural MRI data were downloaded from the ADNI database (*ADSP_PHC_T1_FS* file, accessed March 1, 2026). We used image-derived phenotypes generated by the Alzheimer’s Disease Sequencing Project – Phenotype Harmonization Consortium (ADSP-PHC) [14]. As part of a large-scale harmonization effort, T1-weighted MRI data from ADNI and other AD research cohorts were processed using standardized pipelines to ensure consistency across studies (see ADNI_ADSP_PHC_T1_FS_MUSE_METHODS_20250217.pdf). Raw DICOM files were converted to NIFTI format using either dcm2niix or FreeSurfer’s mri_convert [14–16]. Metadata were preserved in JSON files, and semi-automated workflows were used to standardize filenames, extract metadata, select appropriate T1 scans, and perform quality control checks.

Structural segmentation and parcellation was performed using FreeSurfer version 6.0 recon-all pipeline using the cross-sectional processing stream, followed by visual inspection according to ENIGMA quality control protocols [17]. Scans were rated on a three-point scale (1 = failed, 2 = pass with lower quality, 3 = full pass); scans with ratings of 2 or 3 were retained. Outputs were manually reviewed using MRISnapshot. To reduce scanner-related variability across sites, scanner platforms, and field strengths, FreeSurfer data were harmonized using Longitudinal ComBat [18], which preserved the effects of age, sex, race/ethnicity, and diagnosis while removing unwanted batch effects.

Primary MRI outcomes included a validated AD-related brain signature, a composite of hippocampal volume and cortical thickness in 7 AD-vulnerable regions, as well as 3 separate key AD-related regional metrics: hippocampal volume, entorhinal thickness, and precuneus thickness. The hippocampus and entorhinal cortex are canonical medial temporal lobe targets of early tau pathology. The precuneus was included because it is an early site of Aβ accumulation, a hub of the default mode network, and a region where pain- and AD-related changes overlap [19]. The AD-related brain signature, validated within ADNI [20] and externally [21], is calculated as a weighted average of cortical thickness across 7 regions (entorhinal cortex, middle temporal gyrus, bank of the superior temporal sulcus, superior temporal gyrus, isthmus cingulate, lateral orbitofrontal cortex, medial orbitofrontal cortex) plus hippocampal volume. Hippocampal volume was pre-residualized for intracranial volume.

### Cognitive function

Harmonized cognitive composites were generated by the ADSP-PHC and were downloaded from the ADNI database (*ADSP_PHC_COGN_DATADIC_24* file; accessed March 1, 2026). Cognitive function was measured using the Mini-Mental State Examination (MMSE) and composites labeled as memory and executive function by the ADNI. As described in the ADNI methods material online (*ADNI_Cognition_Methods_Psychometric_Analyses_Oct2022.pdf*), these composites were validated in a bi-factor confirmatory model with good fit (memory and executive function models: CFIs > 0.95, TLIs > 0.95, and RMSEAs < 0.10). Scores from the Rey Auditory Verbal Learning Task, Weschler Memory Scale-Revised Logical Memory I and II, and the word recall portion of the MMSE loaded onto the memory composite. Scores from the Category Fluency Animals, Category Fluency Vegetables, Weschler Adult Intelligence Scale-Revised Digit Symbol, Digit Span Backwards, Trails A and B loaded onto the executive function composite.

### Covariates

Covariates included age, sex, education, and depressive symptoms (Geriatric Depression Scale [GDS] short form score [range = 0–15]) [22] at each visit. All continuous predictors, including depressive symptoms, were z-scored so that model estimates represent standardized effect sizes. To account for baseline differences in AD disease staging, we covaried for baseline Aβ positivity status in main analyses and tau status in sensitivity analyses. Binarization of Aβ and tau status was used rather than treating these as continuous outcomes because it allowed us to pool positivity information across both CSF/PET and PET tau modalities — thereby maximizing sample size — while also avoiding cross-platform measurement variability when treating raw biomarker values as continuous covariates.

For Aβ positivity, we used standardized centiloid cutoffs provided by the ADSP-PHC GAAIN atlas pipeline. Individuals were classified as Aβ-positive if their ADSP-PHC-derived centiloid value exceeded 25 centiloids, the established threshold applied uniformly across all tracers in the ADSP-PHC release [23]. When PET data were unavailable, the CSF Aβ42/Aβ40 ratio derived from the Roche Elecsys cobas e 601 platform was used to classify Aβ positivity, with values below 0.057 indicating Aβ positivity. This cutoff was derived using Youden’s index against amyloid PET visual read in ADNI and achieves approximately 90% concordance with PET classification [19, 20].

For tau positivity, we used PET-derived positivity variables developed by the ADSP-PHC, which applied a Gaussian mixture model (GMM) approach to determine tau positivity thresholds separately for each Braak stage (B1/B2 through B6) and within each tau tracer. The GMM identified the tau-negative distribution within each Braak ROI, and the tau positivity threshold was defined as the mean plus 2.5 standard deviations of that negative distribution [24]. Tau positivity was defined as being positive for regions associated with any Braak stage, assessed using the hierarchical Braak staging schema provided in the ADSP-PHC Level 1 tau dataset. For participants without PET tau data, tau positivity was determined using a CSF p-tau181 cutoff of greater than 27 pg/mL, measured on the Roche Elecsys cobas e 601 platform. This cutoff has been validated against amyloid PET and is consistent across ADNI and BioFINDER cohorts [19].

### Statistical analyses

For descriptive purposes, means and standard deviations or frequencies were calculated for demographic variables. Appropriate tests (t-tests, ANOVA, or χ² tests) were used to compare individuals with and without baseline CP. Participants were grouped into 4 classifications based on baseline status: CP with APOE-ε4 carriership (CP+/ε4+); CP without APOE-ε4 carriership (CP+/ε4-), no CP with APOE-ε4 carriership (CP-/ε4+); and no CP without APOE-ε4 carriership (CP-/ε4-).

For main analyses, linear mixed-effects models were conducted using the lmer function in R (version 4.4.0) [25] to examine whether outcomes of interest (CSF, PET, MRI, cognition) differed as a function of CP and APOE-ε4 status over time. Models included random intercepts for participant, nested within ADNI cohort, and fixed effects of CP status, APOE-ε4 status, time (years since baseline), their two-way interactions, and the three-way CP × APOE-ε4 × time interaction. Random slopes for time were tested but resulted in singular fits across the majority of outcomes, indicating insufficient variance to reliably estimate subject-specific slopes rather than model misspecification; therefore, random intercept-only models were retained. Covariates included baseline age, sex, education, baseline Aβ positivity status (to account for initial disease severity), and time-varying depressive symptoms. Baseline tau positivity status was assessed as a covariate but not included in final models to maintain sample size. Sensitivity analyses, however, revealed the effect sizes (βs) for the primary CP × APOE-ε4 × time interactions were largely preserved when additionally adjusting for baseline tau positivity or using p-tau181 as a continuous covariate. These are not used in primary analyses due to significant loss of sample size, which would reduce statistical power.

The CP × APOE-ε4 interaction term was used to test whether baseline levels of the outcomes differed as a function of the joint presence of CP and APOE-ε4 carriership. The CP × APOE-ε4 × time interaction term was used to test whether the rate of longitudinal change differed as a function of the joint presence of CP and APOE-ε4 carriership.

All continuous predictors were z-scored. As such, model estimates are standardized betas (βs) that can be interpreted as effect sizes. For all analyses, p-values were corrected for multiple comparisons using false discovery rate (FDR) correction applied separately within each modality (CSF, PET, MRI, cognition), as outcomes within a modality share biological relatedness.

## Results

### Participant characteristics

At baseline, 30% of participants (*n* = 452) reported CP and 45% (*n* = 666) had an APOE-ε4 allele. The four CP/APOE-ε4 groups comprised: CP-/ε4- (*n* = 573), CP-/ε4+ (*n* = 468), CP+/ε4- (*n* = 254), and CP+/ε4+ (*n* = 198). Table 1 presents baseline demographic information and biomarker status across groups. Groups did not differ significantly in sex distribution (48–52% male across all groups, χ² = 0.25, *p* = .969). Groups differed significantly in age (F = 7.60, *p* < .001): the CP-/ε4- and CP-/ε4 + groups differed in mean age (M = 72.6 vs. 71.3 years; *p* < .05), and the CP+/ε4- group was older than the CP-/ε4 + group (M = 73.9 vs. 71.3 years; *p* < .05). Groups differed in educational attainment (F = 3.32, *p* = .019), with the CP-/ε4- group having the highest education (M = 16.6 years, SD = 2.49) and the CP+/ε4 + group the lowest (M = 16.1 years, SD = 2.69), though no pairwise comparison survived correction. Groups also differed in depressive symptoms (F = 3.99, *p* = .008), with the CP-/ε4- group reporting the fewest symptoms (M = 1.2, SD = 1.42) and the CP+/ε4- group reporting significantly higher levels (M = 1.5, SD = 1.47; *p* < .05). Differences across the four CP/APOE-ε4 groups in baseline indicators of AD-related disease burden — including MCI prevalence, Aβ positivity, and tau positivity — were driven primarily by APOE-ε4 status. Prevalence of MCI was highest in the CP+/ε4 + group (73%) and lowest in the CP-/ε4- group (40%; χ² = 62.41, *p* < .001). All three remaining groups differed significantly from the CP-/ε4- group, the CP-/ε4 + and CP+/ε4 + groups differed significantly from one another, and CP+/ε4- and CP+/ε4 + groups also differed (ps < 0.05). Similarly, Aβ positivity differed across all groups (χ² = 126.67, *p* < .001), with ε4 carriers markedly more likely to be Aβ-positive (CP-/ε4+: 75%; CP+/ε4+: 71%) than non-carriers (CP-/ε4-: 55%; CP+/ε4-: 35%). All pairs differed significantly except the two ε4 + groups (ps < 0.05). Tau positivity, available in a subset of participants (*n* = 622), was highest among ε4 carriers: CP-/ε4+ (53%) and CP+/ε4+ (63%) compared to CP-/ε4- (18%) and CP+/ε4- (33%; χ² = 87.33, *p* < .001). Both ε4 + groups had significantly higher tau positivity than the CP-/ε4- group (ps < 0.05), while the two CP+ groups and the two ε4- groups did not differ. Supplemental Table 1 presents group means, standard deviations, and omnibus test statistics for all biomarker, imaging, and cognitive outcomes by CP/ε4 group at baseline. Supplemental Table 2 provides information on the average follow-up duration for each study outcome, showing participants provided an average of 3 years of data, with a maximum of 15 study years.

### CSF and PET AD-related biomarkers

Results from models examining how CP and APOE-ε4 status interact to predict baseline levels and longitudinal change in AD-related biomarkers are presented in Table 2. At baseline, CP and APOE-ε4 status did not interact to predict CSF Aβ42/Aβ40, PET Aβ burden, or CSF p-tau181 (ps > 0.05). CP did not predict CSF Aβ42/Aβ40 or CSF p-tau181 at baseline (*p* = .189 and *p* = .726, respectively), and the CP × APOE-ε4 interaction at baseline was also non-significant for both outcomes (*p* = .924 and *p* = .623, respectively). Compared with non-carriers, APOE-ε4 carriers had greater PET Aβ burden (β = 0.72, SE = 0.050, *p* < .001), lower CSF Aβ42/Aβ40 (β = − 0.84, SE = 0.067, *p* < .001), and higher CSF p-tau181 (β = 0.48, SE = 0.050, *p* < .001) at baseline.

**Table 2:** Models examining associations of chronic pain and APOE-ε4 status on Alzheimer’s-related biomarkers

	PET Aβ (*n* = 1271)				CSF Aβ42/Aβ40 (*n* = 420)				CSF *p*-tau181 (*n* = 1306)			
	β	SE	t	*p*	β	SE	t	*p*	β	SE	t	*p*
(Intercept)	**− 0.38**	**0.064**	**-5.95**	**< 0.001**	**0.27**	**0.064**	**4.14**	**< 0.001**	**− 0.18**	**0.049**	**-3.66**	**0.001**
Time	**0.05**	**0.010**	**5.12**	**< 0.001**	**− 0.14**	**0.018**	**-7.86**	**< 0.001**	**0.07**	**0.006**	**12.30**	**< 0.001**
Age at Baseline	**0.17**	**0.026**	**6.79**	**< 0.001**	**− 0.26**	**0.040**	**-6.47**	**< 0.001**	**0.21**	**0.026**	**7.89**	**< 0.001**
Sex (Male)	0.10	0.052	1.89	0.100	0.01	0.081	0.17	0.902	− 0.10	0.052	-1.88	0.112
Education	− 0.03	0.026	-1.28	0.239	0.06	0.039	1.61	0.186	**− 0.07**	**0.026**	**-2.71**	**0.015**
Depressive Symptoms	0.01	0.007	1.98	0.096	0.01	0.017	0.82	0.618	0.01	0.006	2.03	0.085
CP	− 0.06	0.068	-0.81	0.417	0.21	0.131	1.56	0.190	− 0.04	0.068	-0.56	0.726
APOE-ε4	**0.72**	**0.050**	**14.36**	**< 0.001**	**− 0.84**	**0.067**	**-12.45**	**< 0.001**	**0.48**	**0.050**	**9.60**	**< 0.001**
CP*Time	0.02	0.012	1.83	0.100	0.01	0.029	0.29	0.879	0.00	0.008	0.41	0.822
CP*APOE-ε4	0.08	0.086	0.96	0.367	0.02	0.197	0.10	0.924	0.07	0.086	0.77	0.623
APOE-ε4*Time	**0.07**	**0.012**	**5.93**	**< 0.001**	0.02	0.024	0.65	0.690	− 0.00	0.007	-0.23	0.895
CP*APOE-ε4*Time	0.03	0.018	1.68	0.124	**− 0.16**	**0.039**	**-4.15**	**< 0.001**	**0.04**	**0.011**	**3.27**	**0.003**

*Aβ* Amyloid-beta, *CP* Chronic pain

Depressive symptoms were measured using the Geriatric Depression Scale. Models are linear mixed models nested within participant and ADNI cohort. P-values corrected for false discovery rate; bolded values indicate statistically significant associations (*p* < .05)

**Fig. 1:**
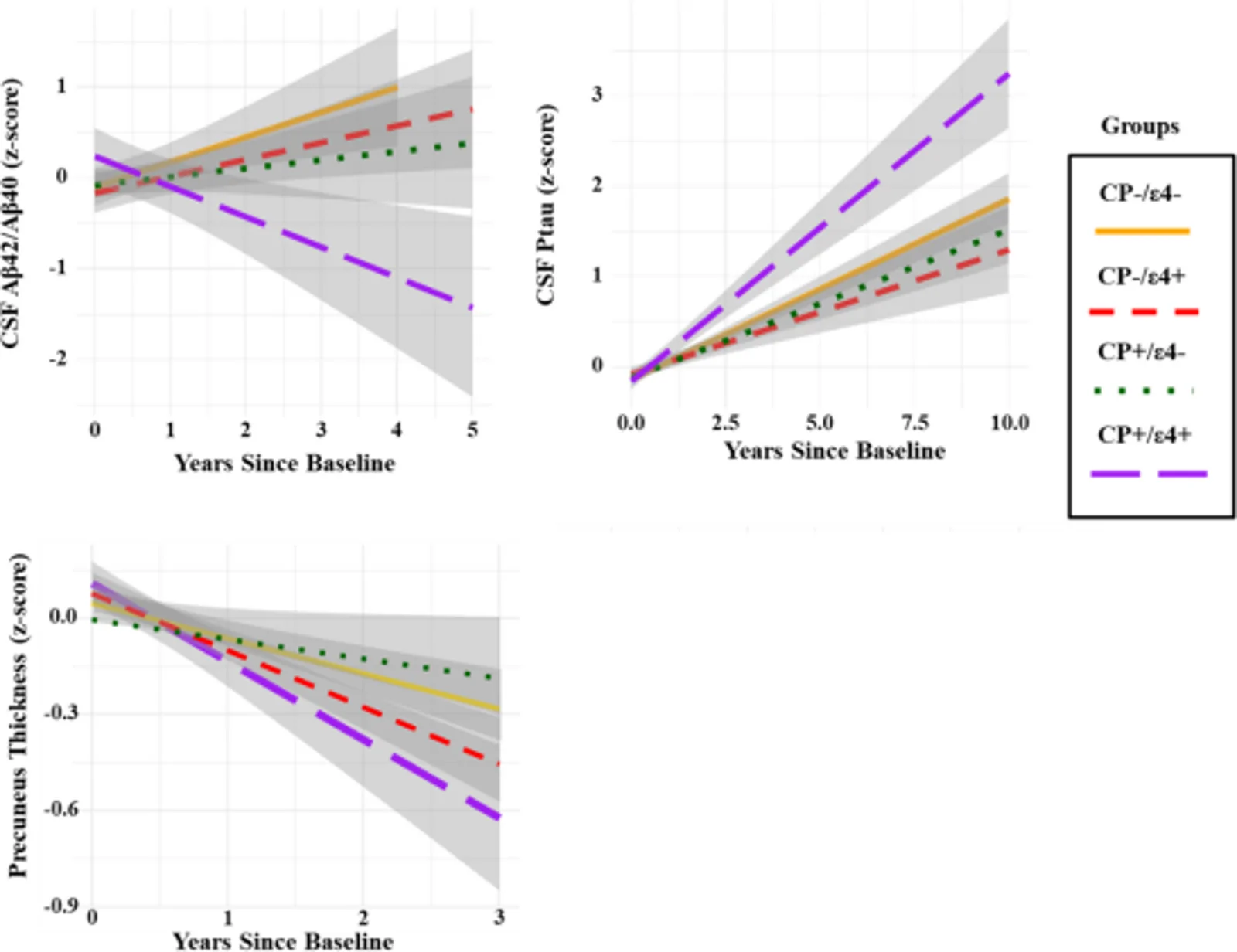
Differences in trajectories of AD-related biomarkers and brain structure across groups classified by baseline chronic pain and APOE-ε4 status. Note. Aβ = amyloid beta; CSF = cerebrospinal fluid; PET = positron emission tomography. Higher PET Aβ, PET Tau, and CSF p-tau-181 are indicative of greater AD pathology. Lower CSF Aβ42/Aβ40 is indicative of greater AD pathology

Longitudinally, CP and APOE-ε4 status did not significantly interact with time to predict change in PET Aβ burden (*p* = .124). However, significant CP × APOE-ε4 × time interactions emerged for CSF Aβ42/Aβ40 (β = − 0.16, SE = 0.039, *p* < .001) and CSF p-tau181 (β = 0.04, SE = 0.011, *p* = .003), indicating that the CP+/ε4 + group showed a steeper decline in CSF Aβ42/Aβ40 and a steeper increase in CSF p-tau181 over time relative to all other groups. For PET Aβ, APOE-ε4 status was independently associated with faster accumulation over time (β = 0.07, SE = 0.012, *p* < .001), while the main effect of CP on PET Aβ accumulation was not significant (*p* = .100). These associations are illustrated in Fig. 1.

Supplemental analyses excluding participants with MCI yielded results largely consistent with the primary analyses (see Supplemental Table 3); no significant three-way interactions were observed for PET Aβ or CSF Aβ42/Aβ40 in the No-MCI subsample, though the CP × APOE-ε4 × time interaction for CSF p-tau181 remained significant (β = 0.10, SE = 0.038, *p* = .025). In the Aβ-negative subsample (see Supplemental Table 6), the CP × APOE-ε4 × time interaction reached significance for PET Aβ accumulation (β = − 0.21, SE = 0.060, *p* = .002), although the direction of this interaction was opposite to that observed in the full sample and its interpretation remains unclear; no three-way interactions were observed for the CSF biomarkers.

### Structural MRI

Results from models examining how CP and APOE-ε4 status interact to predict baseline levels and longitudinal change in AD-related brain regions are presented in Table 3. At baseline, CP and APOE-ε4 status did not interact to predict AD brain signature, hippocampal volume, entorhinal thickness, or precuneus thickness (ps > 0.05). Compared with participants without an APOE-ε4 allele, participants with an APOE-ε4 allele had lower hippocampal volume at baseline (β = − 0.18, SE = 0.047, *p* < .001), but APOE-ε4 was not significantly associated with baseline entorhinal thickness (β = − 0.07, SE = 0.049, *p* = .234).

**Table 3:** Models examining association of chronic pain and APOE-ε4 status with Alzheimer’s-related brain regions

	AD Brain Signature (*n* = 1463)				Hippocampal Volume (*n* = 1447)				Entorhinal Thickness (*n* = 1447)				Precuneus Thickness (*n* = 1447)		
	β	SE	t	*p*	β	SE	t	*p*	β	SE	t	*p*	SE	t	*p*
(Intercept)	− 0.03	0.070	-0.46	0.728	− 0.01	0.089	-0.07	0.961	**0.20**	**0.056**	**3.66**	**0.004**	0.197	0.53	0.719
Time	**− 0.41**	**0.020**	**-20.89**	**< 0.001**	**− 0.15**	**0.007**	**-21.41**	**< 0.001**	**− 0.11**	**0.011**	**-10.23**	**< 0.001**	**0.012**	**-7.93**	**< 0.001**
Age at Baseline	**− 0.20**	**0.016**	**-12.45**	**< 0.001**	**− 0.35**	**0.023**	**-15.57**	**< 0.001**	**− 0.23**	**0.024**	**-9.60**	**< 0.001**	**0.022**	**-11.10**	**< 0.001**
Sex (Male)	**0.19**	**0.031**	**6.01**	**< 0.001**	**0.47**	**0.045**	**10.46**	**< 0.001**	− 0.05	0.047	-0.98	0.472	0.044	-1.34	0.300
Education	0.01	0.016	0.34	0.778	0.02	0.022	0.95	0.472	0.01	0.024	0.30	0.797	0.022	0.73	0.575
Depressive Symptoms	**− 0.07**	**0.013**	**-5.41**	**< 0.001**	− 0.00	0.005	-0.84	0.512	**− 0.02**	**0.008**	**-2.86**	**0.010**	0.008	-0.70	0.587
CP	0.05	0.041	1.33	0.300	− 0.12	0.059	-2.09	0.083	− 0.06	0.063	-0.90	0.477	0.058	1.84	0.131
APOE-ε4	− 0.05	0.033	-1.36	0.300	**− 0.18**	**0.047**	**-3.97**	**< 0.001**	− 0.07	0.049	-1.51	0.234	0.045	-1.80	0.140
Baseline Aβ Positive	**− 0.11**	**0.034**	**-3.10**	**0.005**	**− 0.27**	**0.049**	**-5.43**	**< 0.001**	**− 0.27**	**0.052**	**-5.23**	**< 0.001**	**0.048**	**-3.78**	**< 0.001**
CP*Time	**0.08**	**0.026**	**2.97**	**0.007**	0.01	0.008	0.93	0.472	0.01	0.013	0.93	0.472	0.014	0.00	0.998
CP*APOE-ε4	− 0.05	0.050	-0.97	0.472	0.04	0.075	0.47	0.720	0.03	0.079	0.42	0.728	0.073	-1.05	0.460
APOE-ε4*Time	0.03	0.026	0.96	0.472	**− 0.07**	**0.007**	**-10.35**	**< 0.001**	**− 0.05**	**0.012**	**-3.97**	**< 0.001**	0.013	-2.05	0.083
CP*APOE-ε4*Time	− 0.06	0.037	-1.73	0.155	0.02	0.010	2.07	0.083	− 0.01	0.018	-0.54	0.696	**0.019**	**-4.67**	**< 0.001**

*Aβ* Amyloid-beta, *AD* Alzheimer’s disease, *CP* Chronic pain

Depressive symptoms were measured using the Geriatric Depression Scale. Hippocampal volume pre-residualized for intracranial volume. Models are linear mixed models nested within participant and ADNI cohort. P-values corrected for false discovery rate; bolded values indicate statistically significant associations (*p* < .05)

Longitudinally, CP and APOE-ε4 status interacted to predict change in precuneus thickness. Participants in the CP+/ε4 + group had faster decline in precuneus thickness compared to all other CP/ε4 groups (β = − 0.09, SE = 0.019, *p* < .001). This association is illustrated in Fig. 1. CP and APOE-ε4 status did not interact to predict change in AD brain signature, hippocampal volume, or entorhinal thickness (ps > 0.05; entorhinal CP × APOE-ε4 × time: β = − 0.01, SE = 0.018, *p* = .696). Compared to participants without an APOE-ε4 allele, participants with an APOE-ε4 allele had faster decline in hippocampal volume (β = − 0.07, SE = 0.007, *p* < .001) and faster decline in entorhinal thickness (β = − 0.05, SE = 0.012, *p* < .001), while the association of APOE-ε4 with change in AD brain signature did not reach significance (β = 0.03, SE = 0.026, *p* = .472).

**Fig. 2:**
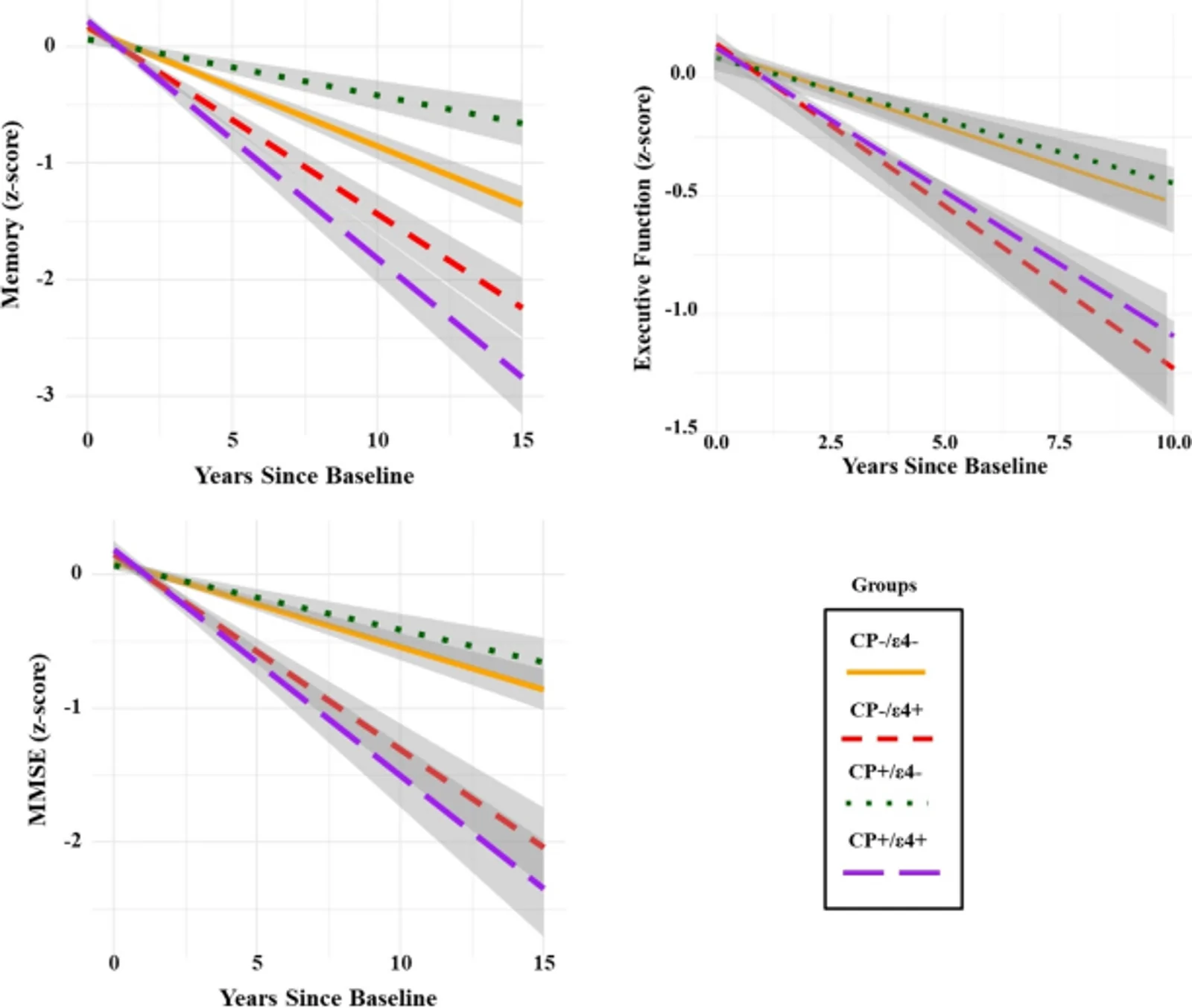
Differences in cognitive trajectories across groups classified by baseline chronic pain and APOE-ε4 status. Note. MMSE = Mini-Mental State Examination. Higher MMSE scores indicate better global cognitive status. Memory and executive function are composite scores, where higher scores indicate better performance

When excluding participants with MCI at baseline (see Supplemental Table 4), the CP × APOE-ε4 × time interaction remained significant for hippocampal volume decline (β = − 0.07, SE = 0.026, *p* = .029), reached significance for the AD brain signature (β = − 0.22, SE = 0.077, *p* = .021), and also reached significance for entorhinal thickness decline (β = − 0.17, SE = 0.054, *p* = .007). The interaction for precuneus thinning did not reach significance in the No-MCI subsample (*p* = .247).

For MRI outcomes in the Aβ-negative subsample (see Supplemental Table 7), a significant CP × APOE-ε4 × time interaction was observed for the AD brain signature (β = − 0.28, SE = 0.071, *p* < .001), indicating that CP+/ε4 + participants showed greater progression on this composite measure even in the absence of baseline Aβ pathology. The interaction for hippocampal volume did not reach significance in the Aβ-negative subsample (*p* = .824), nor did precuneus thickness (*p* = .314).

### Cognitive function

Results from models examining how CP and APOE-ε4 interact to predict baseline levels and longitudinal change in cognition are presented in Table 4. CP and APOE-ε4 status did not interact to predict MMSE, memory, or executive function performance at baseline (ps > 0.05). Compared to participants without an APOE-ε4 allele, participants with an APOE-ε4 allele had worse memory performance at baseline (β = − 0.26, SE = 0.047, *p* < .001).

**Table 4:** Models examining association of chronic pain and APOE-ε4 status with cognition

	MMSE (*n* = 1463)				Memory (*n* = 1463)				Executive Function (*n* = 1463)			
	β	SE	t	*p*	β	SE	t	*p*	β	SE	t	*p*
(Intercept)	**0.28**	**0.075**	**3.74**	**0.010**	**0.58**	**0.061**	**9.48**	**< 0.001**	**0.26**	**0.064**	**4.08**	**0.003**
Time	**− 0.18**	**0.017**	**-10.65**	**< 0.001**	**− 0.11**	**0.010**	**-10.42**	**< 0.001**	**− 0.12**	**0.014**	**-9.25**	**< 0.001**
Age at Baseline	**− 0.11**	**0.021**	**-5.19**	**< 0.001**	**− 0.18**	**0.023**	**-8.06**	**< 0.001**	**− 0.23**	**0.023**	**-10.35**	**< 0.001**
Sex (Male)	**− 0.14**	**0.042**	**-3.37**	**0.001**	**− 0.45**	**0.045**	**-10.02**	**< 0.001**	− 0.09	0.045	-1.91	0.076
Education	**0.16**	**0.021**	**7.72**	**< 0.001**	**0.21**	**0.022**	**9.40**	**< 0.001**	**0.24**	**0.022**	**10.85**	**< 0.001**
Depressive Symptoms	**− 0.04**	**0.012**	**-3.38**	**0.001**	**− 0.02**	**0.007**	**-3.27**	**0.002**	**− 0.03**	**0.009**	**-2.88**	**0.006**
CP	0.10	0.056	1.75	0.104	0.01	0.059	0.12	0.901	0.01	0.059	0.25	0.828
APOE-ε4	**− 0.17**	**0.044**	**-3.92**	**< 0.001**	**− 0.26**	**0.047**	**-5.53**	**< 0.001**	− 0.07	0.046	-1.44	0.189
Baseline Aβ Positive	**− 0.37**	**0.046**	**-7.97**	**< 0.001**	**− 0.47**	**0.049**	**-9.62**	**< 0.001**	**− 0.43**	**0.049**	**-8.73**	**< 0.001**
CP*Time	0.01	0.021	0.57	0.615	**0.03**	**0.012**	**2.85**	**0.007**	0.02	0.016	1.26	0.244
CP*APOE-ε4	− 0.07	0.069	-1.05	0.329	− 0.03	0.075	-0.38	0.742	− 0.10	0.074	-1.34	0.221
APOE-ε4*Time	**− 0.10**	**0.021**	**-4.94**	**< 0.001**	**− 0.08**	**0.012**	**-6.53**	**< 0.001**	− 0.02	0.016	-1.20	0.265
CP*APOE-ε4*Time	**− 0.07**	**0.030**	**-2.35**	**0.026**	**− 0.08**	**0.017**	**-4.82**	**< 0.001**	**− 0.09**	**0.023**	**-3.70**	**< 0.001**

*Aβ* Amyloid-beta, *CP* Chronic pain, *MMSE* Mini-Mental State Examination

Depressive symptoms were measured using the Geriatric Depression Scale. Models are linear mixed models nested within participant and ADNI cohort. P-values corrected for false discovery rate; bolded values indicate statistically significant associations (*p* < .05)

Longitudinally, CP and APOE-ε4 status interacted to predict decline in memory performance. Participants in the CP+/ε4 + group had faster decline in memory compared to all other CP/ε4 groups (β = − 0.08, SE = 0.017, *p* < .001). These associations are illustrated in Fig. 2. CP and APOE-ε4 status also interacted to predict change in MMSE (β = − 0.07, SE = 0.030, *p* = .026), with the CP+/ε4 + group showing faster decline compared to all other groups. The CP × APOE-ε4 × time interaction was also significant for executive function (β = − 0.09, SE = 0.023, *p* < .001), with the CP+/ε4 + group showing faster decline compared to all other groups.

After excluding participants with MCI at baseline, results were largely consistent (see Supplemental Table 5). Longitudinally, participants in the CP+/ε4 + group had significantly faster decline in MMSE (β = − 0.30, SE = 0.073, *p* < .001), memory (β = − 0.31, SE = 0.047, *p* < .001), and executive function (β = − 0.16, SE = 0.054, *p* = .009) compared to all other CP/ε4 groups.

In the Aβ-negative subsample (see Supplemental Table 8), significant CP × APOE-ε4 × time interactions were observed for memory (β = − 0.13, SE = 0.035, *p* < .001) and MMSE (β = − 0.20, SE = 0.059, *p* = .001), with CP+/ε4 + participants showing faster decline on both measures than all other groups. The CP × APOE-ε4 × time interaction was not significant for executive function (β = 0.004, SE = 0.046, *p* = .952) in the Aβ-negative subsample.

## Discussion

CP is a candidate risk factor for AD/ADRD, yet it has not been included in the Lancet Commission’s list of dementia risk factors, highlighting the need for additional evidence [4]. The present study examined whether CP is associated with longitudinal worsening of AD-related biomarkers, brain structure, and cognition, and whether these associations are more pronounced in the presence of APOE-ε4 carriership. Several findings emerged. First, CP and APOE-ε4 status did not independently predict baseline CSF Aβ42/Aβ40, PET Aβ burden, or CSF p-tau181; however, the CP+/ε4 + group showed a significantly steeper longitudinal increase in CSF p-tau181 and decline in CSF Aβ42/Aβ40 compared to all other groups, suggesting that the combination of CP and genetic risk was associated with greater amyloid- and tau-related fluid biomarker change over time. Second, and most consistently, the joint presence of CP and APOE-ε4 carriership was associated with greater longitudinal worsening across multiple outcome domains: greater precuneus thinning, episodic memory decline, and MMSE decline over time compared to all other groups. Third, when restricting analyses to cognitively unimpaired participants, associations extended to entorhinal thickness, hippocampal volume, and the AD brain signature composite. Fourth, in participants who were Aβ-negative at baseline, significant CP × APOE-ε4 × time interactions emerged for the AD brain signature composite, memory, and MMSE, indicating that CP-related vulnerability is not simply a reflection of advanced disease stage. These findings support the hypothesis that CP is associated with a pattern of structural and cognitive vulnerability that is more pronounced in the context of APOE-ε4 carriership, extending our previous ROSMAP findings to an in vivo longitudinal context [8].

We examined CSF and PET Aβ biomarkers and found that individuals with CP who were also APOE-ε4 carriers showed steeper longitudinal change in Aβ-related biomarkers over time, reflected by greater declines in CSF Aβ42/Aβ40 and greater increases in CSF p-tau181, compared with all other groups. Importantly, these differences emerged as longitudinal trajectories rather than baseline differences: neither CP nor APOE-ε4 independently predicted CSF Aβ42/Aβ40 or CSF p-tau181 at baseline, nor did they interact to predict baseline levels. This in vivo evidence of longitudinal amyloid-related and tau-related change in the CP+/ε4 + group aligns with our previous ROSMAP findings of higher postmortem Aβ levels and faster cognitive decline among APOE-ε4 carriers with CP [8]. In ROSMAP, we found no differences in tau tangle density at autopsy, whereas the current study demonstrated steeper increases in CSF p-tau181 over time. This apparent discrepancy may be explained by the fact that CSF p-tau181 reflects soluble phosphorylated tau, whereas postmortem analyses assess aggregated tau burden [26–29]. Evidence suggests that fluid markers of p-tau index early Aβ-related tau hyperphosphorylation and secretion that precede tangle formation [26–29]. Thus, CP and APOE-ε4 carriership may be most strongly associated with early tau dysregulation that has not yet resulted in detectable neurofibrillary pathology at autopsy.

PET Aβ findings, in contrast, revealed only an independent main effect of APOE-ε4, with faster neocortical amyloid accumulation observed among APOE-ε4 carriers regardless of CP status; CP was not independently associated with PET Aβ accumulation and no CP × APOE-ε4 × time interaction was observed. This divergence between CSF and PET biomarkers may reflect the distinct biological processes they capture: CSF Aβ42/Aβ40 indexes soluble Aβ dynamics and clearance, which are strongly influenced by APOE-ε4-related clearance impairment and may be further disrupted by CP-related neuroinflammation, whereas PET Aβ reflects cumulative fibrillar Aβ deposition across the cortex that is driven more dominantly by APOE-ε4 genotype [8]. The absence of a CP × APOE-ε4 interaction on PET Aβ may also reflect a floor effect at this disease stage, in which APOE-ε4-related fibrillar burden is already substantial enough to preclude further differentiation by CP status.

In terms of neurodegeneration, CP and APOE-ε4 carriership did not interact to predict change in the AD brain signature, hippocampal volume, or entorhinal thickness in the full sample. However, individuals with both CP and APOE-ε4 carriership showed significantly greater precuneus thinning over time than all other groups. The precuneus is a hub of the default mode network, a major early site of Aβ accumulation in APOE-ε4 carriers, and a region strongly implicated in pain processing, making it especially vulnerable [30]. When analyses were restricted to cognitively unimpaired participants, the joint association of CP and APOE-ε4 with longitudinal change extended to hippocampal volume decline, entorhinal thickness decline, and the AD brain signature composite, suggesting these effects are not simply driven by the presence of baseline MCI. In the Aβ-negative subsample, a significant CP × APOE-ε4 × time interaction emerged for the AD brain signature, suggesting that CP-related structural vulnerability is not fully dependent on existing Aβ burden and may reflect downstream neurodegeneration that is not fully captured by baseline Aβ measures [31, 32].

Despite no cognitive differences at baseline, individuals with both CP and APOE-ε4 carriership showed significantly greater episodic memory decline over time than all other groups, consistent with our previous work in ROSMAP [8]. When excluding individuals with baseline MCI, this association remained significant and was of larger magnitude, as was the association with MMSE decline. The specificity to memory is consistent with the neuroanatomical pattern of early AD pathology, in which Aβ-induced tauopathy accumulates earliest in medial temporal regions that support episodic memory formation and retrieval [27, 29]. CP and APOE-ε4 also significantly interacted to predict executive function decline, with the CP+/ε4 + group showing faster decline than all other groups. The executive composite used in ADNI comprises primarily processing speed and fluency measures, so this association may reflect broader cognitive vulnerability in CP+/ε4 + adults rather than a frontal-specific effect; conclusions about the specificity of executive decline should be interpreted with this composite structure in mind.

The sensitivity analyses indicate that CP-related differences are not an artifact of pre-existing cognitive impairment or Aβ burden. Associations with memory decline persisted in both the No-MCI and Aβ-negative subsamples. The No-MCI subsample additionally revealed significant interactions for MMSE, executive function, entorhinal thickness, and the AD brain signature. In the Aβ-negative subsample, significant CP × APOE-ε4 × time interactions emerged for the AD brain signature, memory, and MMSE, while hippocampal volume did not reach significance; PET Aβ accumulation also showed a significant three-way interaction in this subsample, but in the opposite direction from the full sample, with the interpretation of this finding remaining unclear. Together, these findings suggest that CP-related vulnerability to neurodegeneration and cognitive decline, particularly in the presence of APOE-ε4, was observed even among participants without detectable pre-existing Aβ or tau burden at baseline. Moreover, the pattern of results was robust to the inclusion of baseline tau positivity as an additional covariate and to the substitution of continuous CSF p-tau181 for binary tau positivity status, with effect sizes remaining in the same range. Given the absence of independent baseline CP main effects on any biomarker, the precise mechanistic role of CP in these associations — whether reflecting initiation of new pathology, amplification of existing pathology, or other modulation of disease trajectories — cannot be determined from observational data and remains unclear. These findings do not establish Aβ-independent mechanisms directly and may still reflect subthreshold pathology or residual confounding. Substantial baseline differences in disease burden across APOE-ε4 groups may also contribute to observed longitudinal differences despite statistical adjustment.

A biologically plausible explanation for this vulnerability is that chronic pain may contribute to a sustained proinflammatory milieu that increases the central nervous system’s sensitivity to AD-related pathology. Peripheral inflammatory signals associated with CP, including elevations in IL-1β, IL-6, and TNF-α, have been shown to influence blood–brain barrier function and promote microglial activation, potentially contributing to a state of heightened neuroinflammatory tone [33, 34]. In such a context, even modest levels of Aβ or tau pathology may have greater downstream effects on synaptic function, neuronal integrity, and network connectivity. APOE-ε4 carriership may further contribute to this vulnerability through impaired Aβ clearance, altered lipid metabolism, and a more reactive microglial profile [35–38]. However, whether CP and APOE-ε4 interact by initiating new pathology, amplifying existing pathology, or otherwise modulating downstream disease processes cannot be determined from observational data and remains unclear. Although these mechanisms are supported by prior experimental and translational work, they were not directly tested in the present study and should be interpreted as a potential explanatory framework rather than a definitive causal pathway.

### Limitations

This study has limitations. Regarding CP phenotyping, CP was classified dichotomously, and information on pain severity, functional impact, number of pain sites, and central versus peripheral pain subtype were not available. This binary classification may not capture heterogeneity and bias effect estimates toward the null, as individuals with mild transient pain may be grouped with those experiencing severe, disabling chronic pain. In addition, this heterogeneity may complicate interpretation of subgroup interactions, as individuals with more severe or multisite pain (who may be at greatest risk) are grouped with those experiencing milder symptoms. Our prior work suggests that moderate-to-severe chronic pain, but not mild pain, is associated with MCI [39], underscoring the importance of pain severity in future studies. Additionally, although individuals using opioids were excluded, we cannot rule out contributions of other pain-related medications such as NSAIDs, antidepressants, or gabapentinoids; however, prior work suggests CP is associated with AD/ADRD risk independent of opioid use [39]. ADNI assesses for the presence of CP at each follow-up visit; however, we focused on baseline CP as a risk factor for subsequent progression rather than modeling changes in pain status over time, which represents a distinct and more complex question that may be influenced by disease-related changes in pain awareness and reporting. Notably, among the 452 individuals classified as CP-positive at baseline, 21 (5%) subsequently reported remission of their pain during follow-up, suggesting that baseline CP status was largely stable and that misclassification due to pain remission is unlikely to substantially bias the present findings. Although our longitudinal design strengthens directional inferences, causality cannot be determined from observational data alone. Reverse directionality also remains plausible: prodromal AD may alter pain perception or reporting through changes in central pain processing networks, potentially inflating the apparent association between CP and AD/ADRD risk. Nonetheless, emerging Mendelian randomization evidence supports a causal effect of multisite CP on cognitive decline and hippocampal atrophy [37].

Chronic pain also frequently co-occurs with cardiovascular disease, diabetes, obesity, and other inflammatory conditions that are independently associated with Aβ accumulation and cognitive decline, raising the possibility that broader multimorbidity may partly explain our findings. Indeed, greater chronic disease burden is associated with faster longitudinal increases in brain Aβ deposition, independent of APOE-ε4 status [40]. However, several considerations mitigate this concern. First, ADNI applies relatively stringent exclusion criteria for major comorbid conditions at enrollment, limiting the degree of multimorbidity in this sample compared to general population cohorts. Second, prior epidemiological studies have found significant associations between CP and dementia risk even after adjusting for cardiovascular disease, diabetes, and other major comorbidities [2, 3], suggesting that the CP-AD association is not fully attributable to shared multimorbidity pathways. Future studies explicitly modeling pain alongside multimorbidity burden will be important to disentangle shared versus condition-specific pathways.

Lastly, inflammatory markers were beyond the scope of the present study but represent promising moderators and/or mediators of the relationship between pain and AD-related biomarkers and neurodegeneration. For example, sTREM2, a marker of microglial activation, may influence tau levels in individuals with CP [7].

## Conclusions

The combination of CP and APOE-ε4 carriership was associated with greater longitudinal worsening across multiple CSF biomarkers, structural MRI measures, and cognitive outcomes, including greater precuneus thinning, memory decline, and MMSE decline compared to all other groups. The CP × APOE-ε4 × time interactions for CSF Aβ42/Aβ40 and CSF p-tau181 further indicate that this synergistic vulnerability extends to the longitudinal trajectory of fluid AD biomarkers. Crucially, associations with memory decline and the AD brain signature persisted among participants who were Aβ-negative at baseline, and entorhinal, hippocampal, and AD brain signature interactions also emerged among cognitively unimpaired participants, indicating that CP-related vulnerability was observed even among individuals classified as Aβ-negative at baseline. These findings add to growing evidence that CP may warrant consideration as a risk factor within dementia prevention frameworks. While this study is observational and cannot establish causality, the findings suggest that CP is associated with greater AD pathology, as well as more pronounced neurodegeneration and cognitive decline among APOE-ε4 carriers. 

## Supplementary Information

Supplementary Material 1.

## Data Availability

Data used in this study are publicly available through the ADNI database (https://ida.loni.usc.edu/).

## Abbreviations

AD/ADRD: Alzheimer’s Disease and Alzheimer’s Disease Related Dementias
AD: Alzheimer’s disease
ADNI: Alzheimer’s Disease Neuroimaging Initiative
Aβ: Amyloid-β
CP: Chronic pain
EF: Executive function
MCI: Mild cognitive impairment
MMSE: Mini-Mental State Examination
MRI: Magnetic resonance imaging
PET: Positron emission tomography
