# Supplementary material for: Chronic pain as a risk factor for Alzheimer’s disease pathology: a longitudinal ADNI study
Source: Alzheimers Res Ther. 2026 Jul 3;18:211. doi: 10.1186/s13195-026-02137-x (PMC13613787; doi:10.1186/s13195-026-02137-x)
Supplement: Supplementary file 1 [file 13195_2026_2137_MOESM1_ESM.docx]

**Supplemental Material**

*Chronic pain as a risk factor for Alzheimer's disease pathology: A longitudinal ADNI study*

**Table of Contents**

| **Table.** | **Page #** |
| --- | --- |
| **Supplemental Table 1.** Descriptive characteristics of participants at baseline by chronic pain and APOE-ε4. | **2** |
| **Supplemental Table 2.** Years of follow-up for study outcomes. | **3** |
| **Supplemental Table 3.** No-MCI sensitivity analysis: Associations of chronic pain and APOE-ε4 status with Alzheimer’s-related biomarkers. | **4** |
| **Supplemental Table 4.** No-MCI sensitivity analysis: Associations of chronic pain and APOE-ε4 status with Alzheimer’s-related brain regions. | **5** |
| **Supplemental Table 5.** No-MCI sensitivity analysis: Associations of chronic pain and APOE-ε4 status with cognitive outcomes. | **6** |
| **Supplemental Table 6.** Aβ-negative sensitivity analysis: Associations of chronic pain and APOE-ε4 status with Alzheimer’s-related biomarkers | **7** |
| **Supplemental Table 7.** Aβ-negative sensitivity analysis: Associations of chronic pain and APOE-ε4 status with Alzheimer’s-related brain regions. | **8** |
| **Supplemental Table 8.** Aβ-negative sensitivity analysis: Associations of chronic pain and APOE-ε4 status with cognitive outcomes. | **9** |

| **Supplemental Table 1. Descriptive characteristics of participants at baseline (continued).** | | | | | | | | |
| --- | --- | --- | --- | --- | --- | --- | --- | --- |
|  |  | Overall | CP−/ε4− | CP−/ε4+ | CP+/ε4− | CP+/ε4+ |  |  |
|  |  | (n = 1493) | (n = 573) | (n = 468) | (n = 254) | (n = 198) | f/χ^2^ | p |
| PET Aβ Burden | M (SD) | 34.8 (46.1) | 16.9 (37.7) | 55.3 (46.0) | 17.8 (38.0) | 61.8 (46.0) | 105.47 | <.001^acdf^ |
|  | n | 1375 | 535 | 430 | 235 | 175 |  |  |
| CSF Aβ42/Aβ40 | M (SD) | 0.064 (0.322) | 0.078 (0.267) | 0.059 (0.309) | 0.089 (0.019) | -0.021 (0.664) | 1.12 | .341 |
|  |  | 487 | 241 | 175 | 33 | 38 |  |  |
| CSF p-tau181 | M (SD) | 0.299 (0.311) | 0.198 (0.240) | 0.392 (0.330) | 0.294 (0.240) | 0.516 (0.425) | 22.46 | <.001^acd^ |
|  | n | 487 | 241 | 175 | 33 | 38 |  |  |
| AD Brain Signature | M (SD) | 638 (176) | 651 (188) | 637 (160) | 635 (173) | 606 (175) | 3.10 | .026^a^ |
|  | n | 1417 | 539 | 450 | 241 | 187 |  |  |
| Hippocampal Volume | M (SD) | 7170 (1050) | 7380 (1030) | 7070 (1050) | 7140 (996) | 6850 (1050) | 14.87 | <.001^acde^ |
|  | n | 1417 | 539 | 450 | 241 | 187 |  |  |
| Entorhinal Thickness | M (SD) | 6.53 (0.886) | 6.66 (0.831) | 6.43 (0.930) | 6.54 (0.865) | 6.39 (0.913) | 7.44 | <.001^ad^ |
|  | n | 1417 | 539 | 450 | 241 | 187 |  |  |
| Precuneus Thickness | M (SD) | 2.25 (0.150) | 2.27 (0.143) | 2.24 (0.160) | 2.25 (0.138) | 2.22 (0.154) | 6.41 | <.001^ad^ |
|  | n | 1417 | 539 | 450 | 241 | 187 |  |  |
| MMSE | M (SD) | 27.7 (2.60) | 28.2 (2.30) | 27.1 (2.91) | 28.1 (2.21) | 27.1 (2.71) | 21.76 | <.001^acdf^ |
|  | n | 1493 | 573 | 468 | 254 | 198 |  |  |
| Memory | M (SD) | 0.337 (0.744) | 0.537 (0.691) | 0.150 (0.789) | 0.432 (0.653) | 0.077 (0.709) | 35.42 | <.001^acdf^ |
|  | n | 1493 | 573 | 468 | 254 | 198 |  |  |
| Executive Function | M (SD) | 0.472 (0.729) | 0.620 (0.681) | 0.362 (0.798) | 0.513 (0.668) | 0.252 (0.670) | 18.34 | <.001^acdf^ |
|  | n | 1493 | 573 | 468 | 254 | 198 |  |  |
| Aβ = amyloid-beta; AD = Alzheimer’s disease; CP = chronic pain; MMSE = Mini-Mental State Examination. Hippocampal volume pre-residualized for intracranial volume. Superscripts denote significant post-hoc pairwise group differences (p <. 05): ^a^ CP−/ε4− vs. CP−/ε4+; ^b^ CP−/ε4− vs. CP+/ε4−; ^c^ CP−/ε4− vs. CP+/ε4+; ^d^ CP−/ε4+ vs. CP+/ε4−; ^e^ CP−/ε4+ vs. CP+/ε4+; ^f^ CP+/ε4− vs. CP+/ε4+. Tukey’s HSD and proportion pairwise tests were used for continuous variables and categorical variables, respectively. | | | | | | | | |

| **Supplemental Table 2. Years of follow-up for study outcomes.** | | |
| --- | --- | --- |
|  | Mean (SD) | Max |
| PET Aβ Burden | 2.48 (2.91) | 12 |
| CSF Aβ42/Aβ40 | .46 (1.63) | 13 |
| CSF p-tau181 | .46 (1.63) | 13 |
| AD Brain Signature | 2.79 (2.94) | 15 |
| Hippocampal Volume | 2.79 (2.94) | 15 |
| Entorhinal Thickness | 2.79 (2.94) | 15 |
| Precuneus Thickness | 2.79 (2.94) | 15 |
| MMSE | 3.27 (3.15) | 15 |
| Memory | 3.28 (3.16) | 15 |
| Executive Function | 3.28 (3.16) | 15 |
| Note. Aβ = amyloid-beta; AD = Alzheimer’s disease; CP = chronic pain; MMSE = Mini-Mental State Examination. Hippocampal volume pre-residualized for intracranial volume. | | |

| **Supplemental Table 3. No-MCI sensitivity analysis: Models examining associations of chronic pain and APOE-ε4 status on Alzheimer's-related biomarkers.** | | | | | | | | | | | | | | |
| --- | --- | --- | --- | --- | --- | --- | --- | --- | --- | --- | --- | --- | --- | --- |
|  | PET Aβ (n = 600) | | | |  | CSF Aβ42/Aβ40 (n = 279) | | | |  | CSF p-tau181 (n = 574) | | | |
|  | β | SE | t | p |  | β | SE | t | p |  | β | SE | t | p |
| (Intercept) | -.21 | .088 | -2.45 | .090 |  | .14 | .076 | 1.80 | .174 |  | -.12 | .097 | -1.25 | .415 |
| Time | **.13** | **.020** | **6.18** | **<.001** |  | **-.14** | **.019** | **-7.32** | **<.001** |  | **.12** | **.015** | **8.43** | **<.001** |
| Age at Baseline | **.17** | **.038** | **4.56** | **<.001** |  | **-.26** | **.050** | **-5.25** | **<.001** |  | **.30** | **.039** | **7.78** | **<.001** |
| Sex (Male) | .02 | .079 | 0.25 | .799 |  | .07 | .104 | 0.71 | .548 |  | -.07 | .078 | -0.88 | .484 |
| Education | -.02 | .039 | -0.63 | .580 |  | .02 | .050 | 0.48 | .660 |  | -.04 | .039 | -1.04 | .450 |
| Depressive Symptoms | .02 | .011 | 1.58 | .198 |  | .02 | .017 | 1.19 | .415 |  | **.04** | **.011** | **3.67** | **.001** |
| CP | -.16 | .103 | -1.61 | .198 |  | **.39** | **.166** | **2.37** | **.049** |  | -.03 | .101 | -0.30 | .762 |
| APOE-ε4 | **.59** | **.081** | **7.32** | **<.001** |  | **-.77** | **.093** | **-8.26** | **<.001** |  | **.28** | **.081** | **3.49** | **.002** |
| Baseline Aβ Positive |  |  |  |  |  |  |  |  |  |  |  |  |  |  |
| CP*Time | -.03 | .024 | -1.05 | .439 |  | .05 | .034 | 1.34 | .394 |  | -.02 | .017 | -1.17 | .415 |
| CP*APOE-ε4 | .17 | .180 | 0.93 | .472 |  | -.27 | .278 | -0.96 | .480 |  | .14 | .165 | 0.87 | .484 |
| APOE-ε4*Time | **.08** | **.025** | **3.10** | **.006** |  | .03 | .027 | 1.13 | .415 |  | -.01 | .017 | -0.52 | .656 |
| CP*APOE-ε4*Time | -.03 | .048 | -0.66 | .580 |  | -.08 | .104 | -0.76 | .541 |  | **.10** | **.038** | **2.64** | **.025** |
| Note. Aβ = amyloid-beta; CP = chronic pain. Depressive symptoms were measured using the Geriatric Depression Scale. Models are linear mixed models nested within participant and ADNI cohort. P-values corrected for false discovery rate; bolded values indicate statistically significant associations (p < .05). | | | | | | | | | | | | | | |

| **Supplemental Table 4. No-MCI sensitivity analysis: Models examining association of chronic pain and APOE-ε4 status with Alzheimer’s-related brain regions.** | | | | | | | | | | | | | | | | | | | |
| --- | --- | --- | --- | --- | --- | --- | --- | --- | --- | --- | --- | --- | --- | --- | --- | --- | --- | --- | --- |
|  | AD Brain Signature (n = 664) | | | |  | Hippocampal Volume (n = 654) | | | |  | Entorhinal Thickness (n = 654) | | | |  | Precuneus Thickness (n = 654) | | | |
|  | β | SE | t | p |  | β | SE | t | p |  |  | β | SE | t | p | β | SE | t | p |
| (Intercept) | -.09 | .071 | -1.21 | .432 |  | -.31 | .112 | -2.75 | .074 |  | .01 | .060 | 0.24 | .895 |  | -.10 | .207 | -0.47 | .762 |
| Time | **-.43** | **.030** | **-14.22** | **<.001** |  | **-.19** | **.012** | **-15.73** | **<.001** |  | **-.16** | **.020** | **-7.96** | **<.001** |  | **-.07** | **.019** | **-3.93** | **<.001** |
| Age at Baseline | **-.14** | **.023** | **-5.92** | **<.001** |  | **-.34** | **.034** | **-10.23** | **<.001** |  | **-.19** | **.034** | **-5.65** | **<.001** |  | **-.26** | **.032** | **-8.21** | **<.001** |
| Sex (Male) | **.18** | **.047** | **3.89** | **<.001** |  | **.63** | **.069** | **9.04** | **<.001** |  | .01 | .071 | 0.08 | .951 |  | -.15 | .065 | -2.33 | .058 |
| Education | .001 | .023 | 0.04 | .969 |  | -.05 | .035 | -1.38 | .324 |  | -.04 | .035 | -1.21 | .371 |  | .02 | .033 | 0.72 | .645 |
| Depressive Symptoms | **-.07** | **.019** | **-3.72** | **<.001** |  | -.01 | .008 | -0.73 | .645 |  | **-.04** | **.016** | **-2.73** | **.023** |  | -.02 | .013 | -1.59 | .253 |
| CP | .10 | .058 | 1.75 | .208 |  | -.12 | .090 | -1.33 | .341 |  | -.05 | .090 | -0.52 | .735 |  | .13 | .086 | 1.51 | .276 |
| APOE-ε4 | .03 | .053 | 0.56 | .728 |  | -.11 | .077 | -1.40 | .321 |  | .08 | .079 | 1.05 | .453 |  | -.01 | .073 | -0.13 | .948 |
| Baseline Aβ Positive | -.04 | .050 | -0.84 | .583 |  | -.03 | .075 | -0.44 | .762 |  | -.10 | .077 | -1.24 | .371 |  | -.04 | .071 | -0.63 | .690 |
| CP*Time | .10 | .042 | 2.34 | .058 |  | .01 | .014 | 0.68 | .665 |  | .03 | .028 | 1.00 | .471 |  | .002 | .023 | 0.09 | .951 |
| CP*APOE-ε4 | -.16 | .097 | -1.68 | .233 |  | .08 | .150 | 0.51 | .735 |  | -.02 | .152 | -0.13 | .948 |  | -.17 | .141 | -1.21 | .371 |
| APOE-ε4*Time | .12 | .048 | 2.40 | .053 |  | -.02 | .015 | -1.20 | .371 |  | -.01 | .030 | -0.35 | .823 |  | .04 | .024 | 1.54 | .267 |
| CP*APOE-ε4*Time | **-.22** | **.077** | **-2.79** | **.021** |  | **-.07** | **.026** | **-2.63** | **.029** |  | **-.17** | **.054** | **-3.18** | **.007** |  | -.07 | .044 | -1.62 | .247 |
| Note. Aβ = amyloid-beta; CP = chronic pain. Depressive symptoms were measured using the Geriatric Depression Scale. Models are linear mixed models nested within participant and ADNI cohort. P-values corrected for false discovery rate; bolded values indicate statistically significant associations (p < .05). | | | | | | | | | | | | | | | | | | | |

| **Supplemental Table 5. No-MCI sensitivity analysis: Models examining association of chronic pain and APOE-ε4 status with cognition.** | | | | | | | | | | | | | | |
| --- | --- | --- | --- | --- | --- | --- | --- | --- | --- | --- | --- | --- | --- | --- |
|  | MMSE (n = 664) | | | |  | Memory (n = 664) | | | |  | Executive Function (n = 664) | | | |
|  | β | SE | t | p |  | β | SE | t | p |  | β | SE | t | p |
| (Intercept) | .18 | .082 | 2.18 | .147 |  | .37 | .121 | 3.03 | .063 |  | .09 | .087 | 1.01 | .474 |
| Time | **-.21** | **.029** | **-7.14** | **<.001** |  | **-.13** | **.021** | **-6.16** | **<.001** |  | **-.20** | **.024** | **-8.41** | **<.001** |
| Age at Baseline | **-.12** | **.027** | **-4.44** | **<.001** |  | **-.28** | **.031** | **-8.98** | **<.001** |  | **-.32** | **.031** | **-10.30** | **<.001** |
| Sex (Male) | **-.22** | **.055** | **-3.97** | **<.001** |  | **-.53** | **.064** | **-8.32** | **<.001** |  | .001 | .063 | 0.01 | .989 |
| Education | **.12** | **.027** | **4.32** | **<.001** |  | **.22** | **.032** | **6.88** | **<.001** |  | **.23** | **.031** | **7.17** | **<.001** |
| Depressive Symptoms | -.01 | .019 | -0.46 | .761 |  | -.01 | .014 | -0.55 | .706 |  | -.01 | .016 | -0.72 | .616 |
| CP | .06 | .071 | 0.80 | .573 |  | .09 | .083 | 1.05 | .424 |  | .11 | .082 | 1.37 | .276 |
| APOE-ε4 | .03 | .063 | 0.56 | .706 |  | .02 | .071 | 0.22 | .895 |  | -.01 | .070 | -0.16 | .919 |
| Baseline Aβ Positive | -.10 | .059 | -1.66 | .172 |  | -.15 | .069 | -2.21 | .063 |  | **-.18** | **.068** | **-2.66** | **.021** |
| CP*Time | -.01 | .040 | -0.22 | .895 |  | .04 | .026 | 1.47 | .240 |  | .05 | .030 | 1.75 | .158 |
| CP*APOE-ε4 | -.20 | .116 | -1.70 | .168 |  | -.27 | .137 | -1.94 | .114 |  | -.17 | .134 | -1.28 | .302 |
| APOE-ε4*Time | .01 | .044 | 0.13 | .922 |  | -.01 | .029 | -0.33 | .851 |  | .04 | .033 | 1.34 | .281 |
| CP*APOE-ε4*Time | **-.30** | **.073** | **-4.03** | **<.001** |  | **-.31** | **.047** | **-6.57** | **<.001** |  | **-.16** | **.054** | **-2.94** | **.009** |
| Note. Aβ = amyloid-beta; CP = chronic pain. Depressive symptoms were measured using the Geriatric Depression Scale. Models are linear mixed models nested within participant and ADNI cohort. P-values corrected for false discovery rate; bolded values indicate statistically significant associations (p < .05). | | | | | | | | | | | | | | |

| **Supplemental Table 6. Aβ-negative sensitivity analysis: Models examining associations of chronic pain and APOE-ε4 status on Alzheimer's-related biomarkers.** | | | | | | | | | | | | | | |
| --- | --- | --- | --- | --- | --- | --- | --- | --- | --- | --- | --- | --- | --- | --- |
|  | PET Aβ (n = 640) | | | |  | CSF Aβ42/Aβ40 (n = 240) | | | |  | CSF p-tau181 (n = 635) | | | |
|  | β | SE | t | p |  | β | SE | t | p |  | β | SE | t | p |
| (Intercept) | -.20 | .144 | -1.41 | .267 |  | .14 | .106 | 1.31 | .388 |  | -.06 | .064 | -0.94 | .562 |
| Time | .03 | .026 | 1.32 | .251 |  | **-.21** | **.040** | **-5.39** | **<.001** |  | **.07** | **.010** | **7.02** | **<.001** |
| Age at Baseline | **-.10** | **.037** | **-2.74** | **.011** |  | **-.18** | **.055** | **-3.20** | **.005** |  | **.29** | **.038** | **7.70** | **<.001** |
| Sex (Male) | **.20** | **.073** | **2.77** | **.011** |  | .07 | .112 | 0.67 | .757 |  | .02 | .074 | 0.29 | .881 |
| Education | **.10** | **.037** | **2.75** | **.011** |  | .02 | .054 | 0.37 | .881 |  | -.01 | .037 | -0.20 | .881 |
| Depressive Symptoms | -.00 | .019 | -0.09 | .975 |  | .06 | .034 | 1.80 | .147 |  | .02 | .010 | 1.54 | .230 |
| CP | -.00 | .091 | -0.03 | .975 |  | .05 | .169 | 0.29 | .881 |  | -.00 | .088 | -0.03 | .974 |
| APOE-ε4 | **.30** | **.102** | **2.93** | **.011** |  | **-.70** | **.137** | **-5.10** | **<.001** |  | .20 | .108 | 1.84 | .146 |
| CP*Time | **.11** | **.030** | **3.78** | **.001** |  | .01 | .054 | 0.20 | .881 |  | **.06** | **.012** | **5.02** | **<.001** |
| CP*APOE-ε4 | -.32 | .183 | -1.76 | .119 |  | -.11 | .372 | -0.30 | .881 |  | .40 | .178 | 2.27 | .071 |
| APOE-ε4*Time | **.29** | **.041** | **6.98** | **<.001** |  | -.12 | .060 | -1.94 | .129 |  | **.10** | **.017** | **5.57** | **<.001** |
| CP*APOE-ε4*Time | **-.21** | **.060** | **-3.53** | **.002** |  | -.16 | .080 | -1.97 | .129 |  | .01 | .023 | 0.54 | .833 |
| Note. Aβ = amyloid-beta; CP = chronic pain. Depressive symptoms were measured using the Geriatric Depression Scale. Models are linear mixed models nested within participant and ADNI cohort. P-values corrected for false discovery rate; bolded values indicate statistically significant associations (p < .05). | | | | | | | | | | | | | | |

| **Supplemental Table 7. Aβ-negative sensitivity analysis: Models examining association of chronic pain and APOE-ε4 status with Alzheimer’s-related brain regions.** | | | | | | | | | | | | | | | | | | | |
| --- | --- | --- | --- | --- | --- | --- | --- | --- | --- | --- | --- | --- | --- | --- | --- | --- | --- | --- | --- |
|  | AD Brain Signature (n = 747) | | | |  | Hippocampal Volume (n = 738) | | | |  | Entorhinal Thickness (n = 738) | | | |  | Precuneus Thickness (n = 738) | | | |
|  | β | SE | t | p |  | β | SE | t | p |  |  | β | SE | t | p | β | SE | t | p |
| (Intercept) | -.10 | .059 | -1.71 | .311 |  | -.17 | .074 | -2.27 | .125 |  | .04 | .054 | 0.74 | .738 |  | .02 | .189 | 0.08 | .956 |
| Time | **-.43** | **.027** | **-16.20** | **<.001** |  | **-.14** | **.009** | **-14.58** | **<.001** |  | **-.09** | **.013** | **-7.03** | **<.001** |  | **-.05** | **.017** | **-2.84** | **.016** |
| Age at Baseline | **-.22** | **.021** | **-10.08** | **<.001** |  | **-.43** | **.031** | **-13.82** | **<.001** |  | **-.21** | **.033** | **-6.53** | **<.001** |  | **-.28** | **.031** | **-9.21** | **<.001** |
| Sex (Male) | **.16** | **.042** | **3.85** | **<.001** |  | **.44** | **.062** | **7.08** | **<.001** |  | -.08 | .065 | -1.18 | .455 |  | -.09 | .061 | -1.44 | .314 |
| Education | -.01 | .021 | -0.53 | .824 |  | .02 | .031 | 0.80 | .702 |  | .01 | .033 | 0.27 | .909 |  | .06 | .031 | 1.98 | .137 |
| Depressive Symptoms | **-.08** | **.018** | **-4.73** | **<.001** |  | .001 | .007 | 0.09 | .956 |  | -.01 | .012 | -0.98 | .563 |  | -.01 | .013 | -1.01 | .563 |
| CP | .05 | .049 | 1.00 | .563 |  | -.14 | .075 | -1.84 | .168 |  | -.03 | .078 | -0.45 | .873 |  | .02 | .074 | 0.29 | .909 |
| APOE-ε4 | .03 | .063 | 0.54 | .824 |  | -.01 | .089 | -0.12 | .956 |  | .11 | .094 | 1.22 | .447 |  | -.03 | .087 | -0.39 | .905 |
| CP*Time | **.09** | **.035** | **2.52** | **.038** |  | .004 | .010 | 0.36 | .909 |  | .01 | .017 | 0.71 | .740 |  | **-.06** | **.019** | **-3.39** | **.003** |
| CP*APOE-ε4 | -.14 | .099 | -1.45 | .314 |  | -.10 | .152 | -0.66 | .764 |  | -.04 | .160 | -0.26 | .909 |  | -.03 | .148 | -0.23 | .909 |
| APOE-ε4*Time | **.24** | **.055** | **4.27** | **<.001** |  | -.005 | .014 | -0.33 | .909 |  | -.05 | .025 | -1.91 | .149 |  | .002 | .027 | 0.09 | .956 |
| CP*APOE-ε4*Time | **-.28** | **.071** | **-3.91** | **<.001** |  | -.01 | .019 | -0.52 | .824 |  | -.00 | .033 | -0.01 | .996 |  | -.05 | .036 | -1.48 | .314 |
| Note. Aβ = amyloid-beta; CP = chronic pain. Depressive symptoms were measured using the Geriatric Depression Scale. Models are linear mixed models nested within participant and ADNI cohort. P-values corrected for false discovery rate; bolded values indicate statistically significant associations (p < .05). | | | | | | | | | | | | | | | | | | | |

| **Supplemental Table 8. Aβ-negative sensitivity analysis: Models examining association of chronic pain and APOE-ε4 status with cognition.** | | | | | | | | | | | | | | |
| --- | --- | --- | --- | --- | --- | --- | --- | --- | --- | --- | --- | --- | --- | --- |
|  | MMSE (n = 747) | | | |  | Memory (n = 747) | | | |  | Executive Function (n = 747) | | | |
|  | β | SE | t | p |  | β | SE | t | p |  | β | SE | t | p |
| (Intercept) | .11 | .062 | 1.73 | .178 |  | **.36** | **.076** | **4.75** | **.001** |  | .07 | .073 | 1.00 | .453 |
| Time | **-.13** | **.023** | **-5.73** | **<.001** |  | **-.06** | **.016** | **-3.85** | **<.001** |  | **-.07** | **.019** | **-3.53** | **.001** |
| Age at Baseline | **-.14** | **.029** | **-4.94** | **<.001** |  | **-.22** | **.032** | **-6.83** | **<.001** |  | **-.23** | **.031** | **-7.27** | **<.001** |
| Sex (Male) | **-.21** | **.056** | **-3.80** | **<.001** |  | **-.61** | **.063** | **-9.80** | **<.001** |  | -.11 | .061 | -1.80 | .131 |
| Education | **.20** | **.028** | **7.16** | **<.001** |  | **.26** | **.032** | **8.18** | **<.001** |  | **.29** | **.031** | **9.40** | **<.001** |
| Depressive Symptoms | **-.07** | **.017** | **-3.77** | **<.001** |  | **-.04** | **.011** | **-3.61** | **<.001** |  | **-.04** | **.014** | **-2.50** | **.025** |
| CP | .07 | .068 | 0.96 | .453 |  | -.01 | .076 | -0.16 | .926 |  | .03 | .074 | 0.42 | .842 |
| APOE-ε4 | -.02 | .083 | -0.22 | .926 |  | -.02 | .090 | -0.22 | .926 |  | .05 | .089 | 0.52 | .776 |
| CP*Time | -.001 | .029 | -0.04 | .965 |  | .02 | .017 | 1.10 | .391 |  | -.03 | .022 | -1.41 | .250 |
| CP*APOE-ε4 | -.17 | .136 | -1.23 | .328 |  | -.26 | .153 | -1.67 | .162 |  | -.31 | .150 | -2.09 | .070 |
| APOE-ε4*Time | **.18** | **.046** | **3.95** | **<.001** |  | -.01 | .027 | -0.28 | .926 |  | -.01 | .036 | -0.18 | .926 |
| CP*APOE-ε4*Time | **-.20** | **.059** | **-3.44** | **.001** |  | **-.13** | **.035** | **-3.58** | **<.001** |  | .004 | .046 | 0.09 | .952 |
| Note. Aβ = amyloid-beta; CP = chronic pain. Depressive symptoms were measured using the Geriatric Depression Scale. Models are linear mixed models nested within participant and ADNI cohort. P-values corrected for false discovery rate; bolded values indicate statistically significant associations (p < .05). | | | | | | | | | | | | | | |
